# Airflow attenuation and bed net utilization: observations from Africa and Asia

**DOI:** 10.1186/1475-2875-11-200

**Published:** 2012-06-15

**Authors:** Lorenz von Seidlein, Konstantin Ikonomidis, Rasmus Bruun, Musa Jawara, Margaret Pinder, Bart GJ Knols, Jakob B Knudsen

**Affiliations:** 1 Menzies School of Health Research, John Mathews Building (Bldg 58), PO Box 41096, Casuarina, NT, 0810, Australia; 2 Det Kongelige Danske Kunstakademis Skoler for Arkitektur, Design og Konservering – Arkitektskolen, Copenhagen, Denmark; 3 The Medical Research Council Laboratories, Fajara, The Gambia; 4 London School Hygiene and Tropical Medicine, London, UK; 5 In2Care BV, Wageningen, The Netherlands

**Keywords:** Malaria, Bed nets, Africa, Tanzania, The Gambia, Asia, Thailand, Philippines, Airflow, Temperature, Humidity, Climate

## Abstract

**Background/Methods:**

Qualitative studies suggest that bed nets affect the thermal comfort of users. To understand and reduce this discomfort the effect of bed nets on temperature, humidity, and airflow was measured in rural homes in Asia and Africa, as well as in an experimental wind tunnel. Two investigators with architectural training selected 60 houses in The Gambia, Tanzania, Philippines, and Thailand. Data-loggers were used to measure indoor temperatures in hourly intervals over a 12 months period. In a subgroup of 20 houses airflow, temperature and humidity were measured at five-minute intervals for one night from 21.00 to 6.00 hrs inside and outside of bed nets using sensors and omni-directional thermo-anemometers. An investigator set up a bed net with a mesh size of 220 holes per inch^2^ in each study household and slept under the bed net to simulate a realistic environment. The attenuation of airflow caused by bed nets of different mesh sizes was also measured in an experimental wind tunnel.

**Results:**

The highest indoor temperatures (49.0 C) were measured in The Gambia. During the hottest months of the year the mean temperature at night (9 pm) was between 33.1 C (The Gambia) and 26.2 C (Thailand). The bed net attenuated the airflow from a minimum of 27% (Philippines) to a maximum of 71% (The Gambia). Overall the bed nets reduced airflow compared to un-attenuated airflow from 9 to 4 cm sec^-1^ or 52% (p < 0.001). In all sites, no statistically significant difference in temperature or humidity was detected between the inside and outside of the bed net. Wind tunnel experiments with 11 different mesh-sized bed nets showed an overall reduction in airflow of 64% (range 55 - 71%) compared to un-attenuated airflow. As expected, airflow decreased with increasing net mesh size. Nets with a mesh of 136 holes inch^-2^ reduced airflow by 55% (mean; range 51 - 73%). A denser net (200 holes inch^-2^) attenuated airflow by 59% (mean; range 56 - 74%).

**Discussion:**

Despite concerted efforts to increase the uptake of this intervention in many areas uptake remains poor. Bed nets reduce airflow, but have no influence on temperature and humidity. The discomfort associated with bed nets is likely to be most intolerable during the hottest and most humid period of the year, which frequently coincides with the peak of malaria vector densities and the force of pathogen transmission.

**Conclusions:**

These observations suggest thermal discomfort is a factor limiting bed net use and open a range of architectural possibilities to overcome this limitation.

## Background

A meta-analysis by Lengeler found that insecticide treated bed nets (ITNs) afford a 17% protection against mortality in children under five years of age [1]. ITNs also reduce the incidence of mild malaria by 48% compared to no net and by 34% compared to non-impregnated nets [1]. The international donor community is investing large amounts of money to make ITN’s available in malaria-endemic regions. Between 2006 and 2008 nearly 140 million ITNs were distributed in the African Region where approximately 671 million people are at risk of malaria [2]. Nevertheless, to many public health experts who promote this intervention, the observed uptake has been disappointingly low. In Togo and Sierra Leone, for instance, following mass distribution of ITNs, ownership dropped by 13% and 37% within 24 to 36 months, respectively [2]. In a study in Niger, as few as 33% of the net owners had used their net the night prior to the interview [3]. Recent work by Trape and co-workers in Senegal suggests a rebound in malaria incidence after an original positive impact by long-lasting insecticide-impregnated bed nets [4]. ITNs can only protect when they are correctly used throughout the whole night while mosquitoes are host seeking otherwise they afford only a fraction of their potential protection. Pulford and co-workers reviewed self-reported reasons for not using a bed net. In the 22 studies included in their review, the most widely identified reason for non-use of bed nets was discomfort, primarily due to heat [5].

**Figure 1:**
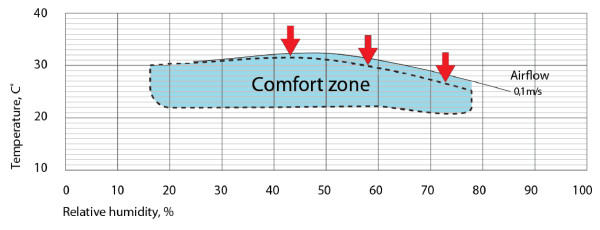
**The comfort zone.** Airflow extends the comfort zone. The red arrows indicate the reduction in the comfort zone if airflow is decreased. At the upper edge of the comfort zone even a subtle reduction in airflow can result in a climate that is intolerable for most people (after Koenigsberger [6]).

Thermal comfort depends on three principal variables, temperature, humidity and airflow. Air-conditioning, which is non-existent or unaffordable for the majority of the population in malaria-endemic resource-poor regions, can regulate temperature and humidity. Airflow can be generated by fans or increased by opening ventilation areas. Koenigsberger [6] defined the thermal zone within which humans feel comfortable based on temperature, humidity and airflow (Figure 1). The model was extended by Ole Fanger who added the parameters of metabolism and clothing to define the individual comfort zone [7]. The models help to understand that subtle changes in airflow at the upper edge of the comfort zone determine the tolerability of indoor climate. In extremely hot and humid conditions even a small attenuation of airflow is sufficient to render a space uncomfortable if not unbearable.

Previous studies have suggested that bed nets affect the thermal comfort of the users [5]. To get a better understanding of the sources of this discomfort the effect of bed nets on temperature, humidity, and airflow was quantified in rural homes in Asia and Africa, as well as in an experimental wind tunnel.

## Methods

### Study sites

**Figure 2:**
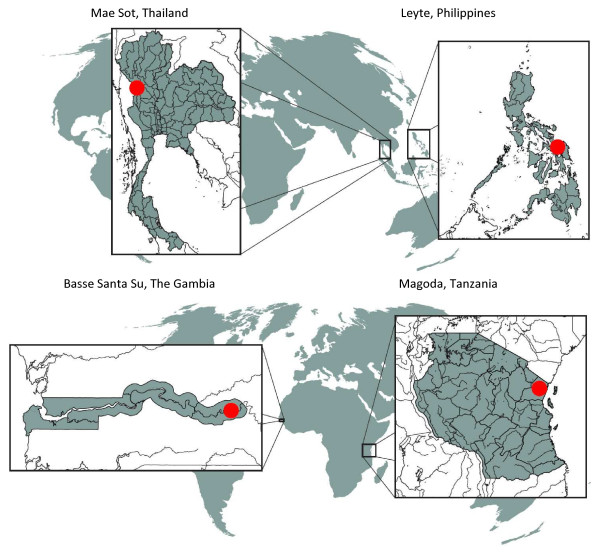
Study locations.

**Figure 3:**
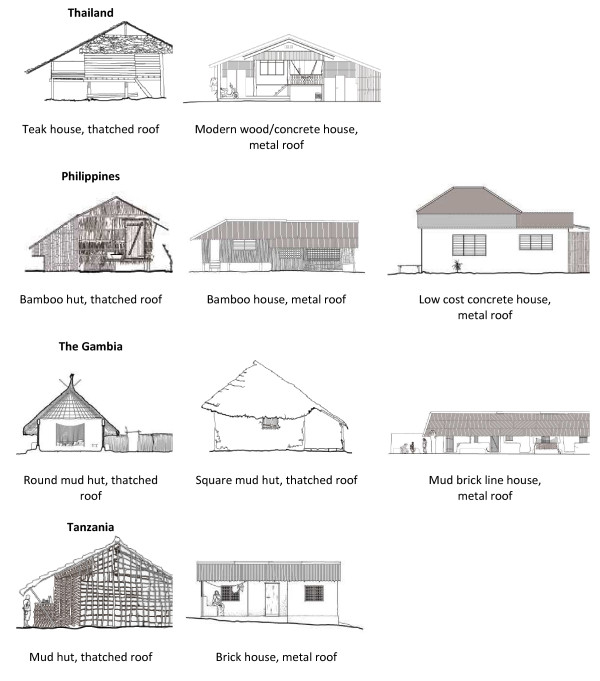
Line drawings of the houses categories in which data were collected.

Four study sites in Asia and Africa were selected based on recommendations from collaborators. In Asia the studies were conducted in Mae Sot, north-western Thailand and in Basud, Leyte Island, in the Visayas region of the Philippines. In Africa, the studies were conducted in rural villages near Basse Santa Su in the Upper River Region of The Gambia and in Magoda village in the Tanga region of Tanzania (Figure 2). Two architecture-trained investigators visited each of the study sites and assessed the general building types present in each location. The investigators selected 15 study houses in each of the 4 study sites to record indoor temperature and humidity in hourly intervals over a 12 months period. In a subgroup of 20 houses (see Additional file 1) airflow, temperature and humidity were measured at five-minute intervals from 21.00 to 6.00 hrs inside and outside of bed nets using sensors and omni-directional thermo-anemometers. The house categories are illustrated in Figure 3.

### Equipment

Hourly measurements of indoor temperature and humidity were obtained by HOBO data loggers over a one year period (ONSET, Bourne, MA, USA). Airflow, temperature, and humidity were measured in- and outside of bed nets over one night within the houses using highly sensitive thermo- (FTA15P, Ahlborn, Germany) and humidity-sensors (FHA646-E1, Ahlborn, Germany) and an omni-directional hot wire thermo-anemometers (ThermoAir 6/64; Schiltknecht, Gossau, Switzerland) connected to a data logger (Almemo 2590–2, Ahlborn, Germany). The calibrated, omni-directional thermo-anemometers, which measure airflow accurately at very low velocity down to 1.5 cm sec^-1^ were used for measurements in the field sites and the wind tunnel.

### Study procedures

Two Hobo data loggers were installed in the room used by the household head for sleeping. The data loggers were placed in a part of the room where they were likely to be undisturbed for a 12 months period. One data logger was placed about 50 cm above ground the other 150 cm above ground.

**Figure 4:**
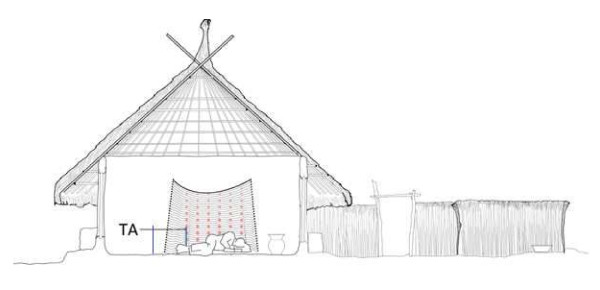
**Set-up for measuring airflow under a bed net inside a home in The Gambia.** One thermo-anemometer (TA) is placed (at 50 cm above ground level) under the bed net and a second thermo-anemometer is placed outside the net at the same height.

The investigators set up a bed net (mesh size 220 holes inch-2), which was purchased in a market in Thailand and used for the airflow measurements in study houses in the four sites. In each study household one investigator slept under the bed net to simulate a realistic environment. Two sets of sensors recorded temperature, humidity and airflow at five-minute intervals. One set of sensors was positioned inside the bed net and the second set outside both at the same level 50 cm above ground level (Figure 4). Data from the sensors “inside/outside” were recorded from 21:00 to 6:00 hrs and compared. To estimate the overall effect of a bed net on the airflow in a room the bed net was removed at three-minute intervals and the airflow was measured every 30 seconds with one set of sensors positioned on the bed. In order to allow the airflow to stabilize between each operation no measurements were taken during a one-minute interval between each change in setup. Comparative airflow during intermittent bed net use (“on/off”) was assessed in four homes, whereas continuous measurements (“inside/outside”) were collected from 16 homes (Table 1).

**Table 1:** airflow measurements in 20 houses in Thailand, Philippines, The Gambia and Tanzania using a bed net with a mesh of 220 holes per square inch

	**#**	**Bed net**	**Median airspeed outside net**	**Median airspeed inside net**	**Attenuation of airflow**	**Median temp**^**†**^**(21:00 to 6:00)**^**0**^ **C**	**Median relative humidity**^**†**^**(21:00 to 6:00)**
**Thailand**	1	On/off	0.14	0.06	57%	25.5	43%
	2	On/off	0.11	0.06	45%	24.5	55%
	3	On/off	0.15	0.1	33%	24.2	67%
**Philippines**	4	On/off	0.14	0.06	57%	25.4	89%
	5	Inside/outside	0.06	0.03	50%	25.5	92%
	6	Inside/outside	0.08	0.03	63%	25.9	92%
	7	Inside/outside	0.11	0.08	27%	24.7	92%
	8	Inside/outside	0.09	0.03	67%	25.1	92%
	9	Inside/outside	0.09	0.06	33%	27.1	90%
**The Gambia**	10	Inside/outside	0.07	0.02	71%	26.3	66%
	11	Inside/outside	0.07	0.02	71%	28.4	62%
	12	Inside/outside	0.09	0.03	67%	25.1	58%
	13	Inside/outside	0.06	0.03	50%	27.0	50%
	14	Inside/outside	0.05	0.03	40%	28.1	56%
	15	Inside/outside	0.09	0.03	67%	26.0	61%
	16	Inside/outside	0.09	0.03	67%	24.3	57%
**Tanzania**	17	Inside/outside	0.06	0.04	33%	26.8	73%
	18	Inside/outside	0.12	0.06	50%	27.7	69%
	19	Inside/outside	0.14	0.08	43%	27.0	73%
	20	Inside/outside	0.08	0.02	75%	28.0	69%
**Overall**			0.09	0.04	50%	26.0	70%

†No significant difference in temperature or humidity was detected inside and outside the net. (Note that the measurements were not conducted during the hottest period of the year); * p-value <0.001 comparing airspeed inside and outside bed net (Mann–Whitney two-sample statistic or Wilcoxon matched-pairs signed-ranks test).

**Figure 5:**
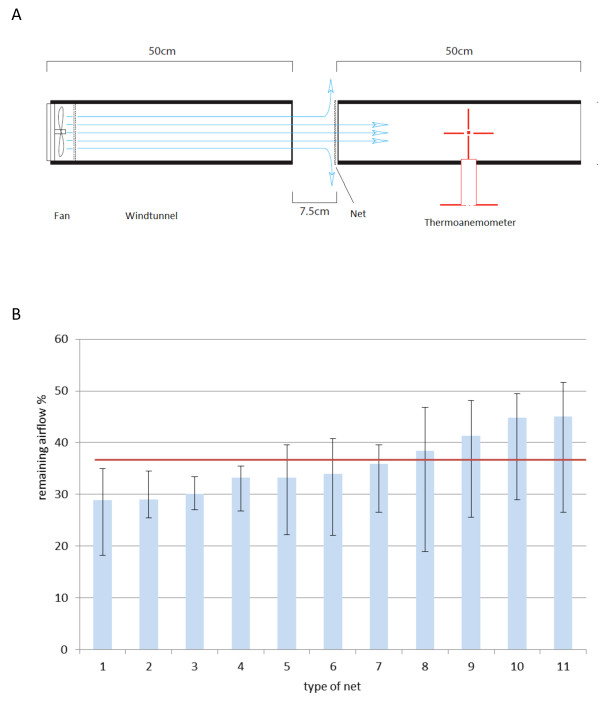
**Experimental set up to measure the attenuation of airflow by nets in a wind tunnel. A:** a fan is placed in a tube with a diameter of 100 mm to direct the airflow. The air flows through a second pipe in which a thermo-anemometer is placed to record the airflow. The airspeed (9, 14, 18, 24, 29, and 36 cm sec^-1^) is measured at 6 different voltage settings. After recording airspeed in the absence of nets, the airspeed was measured after the airflow was attenuated by nets. **B:** Findings from wind tunnel experiments. The remaining airflow behind eleven bed nets at six different air speeds (9, 14, 18, 24, 29, and 36 cm sec^-1^) was measured. The blue bars indicate the mean attenuation in airflow; the error bars indicate the range at the different airspeeds and the red line the average remaining airflow for all 11 bed nets (36%). (Sources of the bed nets: 1, 5, 6 - Philippines; 2 – Thailand; 3, 8 - The Gambia; 4, 7, 10 – Tanzania; 9, 11- Europe).

The attenuation of airflow by nets was experimentally measured in a wind tunnel. An electric fan (diameter 80 mm) was mounted at one end of one section of a PVC pipe 50 cm long with a diameter of 200 mm. A thermo-anemometer was placed inside an identical second section of the pipe (Figure 5A) placed in front of the first pipe with a gap between the pipes. Bed nets were placed in the gap between the two pipe sections. In the absence of bed nets the airspeed was 9, 14, 18, 24, 29, and 36 cm sec^-1^ at 6 different voltage settings. 100 airflow measurements were obtained for each bed net at each voltage setting.

### Bed nets

One net purchased in a market in Thailand (220 holes per inch^2^) was used for all airflow measurements in the four study sites in Asia and Africa. For the wind tunnel experiments conducted in Copenhagen, Denmark 11 bed nets were used. Two widely used long-lasting impregnated bed nets with mesh sizes of 136 and 200 holes per inch^2^ were provided by an industrial bed net manufacturer (Bestnet A/S, Esbjergvej 16A, DK-6000 Kolding Denmark). Nine additional bed nets were purchased in local markets in Thailand (n = 1), the Philippines (n = 3), Tanzania (n = 3), and The Gambia (n = 2).

### Data management and analysis

Excel spread sheets (Microsoft Corp. Redmond, WA 98052–7329, USA) were used to process the data recorded on data loggers connected to each instrument and Stata 11 (StataCorp, College Station, Texas 77845 USA) was used for the statistical analysis. The indoor temperatures recorded over a 12 months period by HOBO data loggers were plotted for each house and inspected. The hottest months of the year were identified for each site as the study period for the analysis. For The Gambia data from February through June were included in the analysis of indoor temperatures, in Tanzania January through April, in the Philippines April through December, and in Thailand April through October. The maximum temperature for each study house and each day during the study period was established and the mean daily maximum temperature for all houses in each site over the study period was calculated. To estimate the “bed time temperature” the daily temperature measurement closest to 21:00 was selected for each study house and the mean temperature for all study houses over the study period was calculated. The wind tunnel experiments were analysed by calculating the means and 95% confidence intervals for each net over a range of voltage related air speeds.

Mann–Whitney two-sample statistics was used to compare the on-off measurements and Wilcoxon matched-pairs signed-ranks test were used for inside/outside comparisons. The attenuation was calculated as 1-proportion of airflow reduction. The discomfort due to the climate under the bed net is expressed as the percentage of people dissatisfied (ppd), which is the internationally accepted the indoor climate standard proposed by the American Society of Heating, Refrigerating and Air-Conditioning Engineers (ASHRAE) [8].

## Results

**Figure 6:**
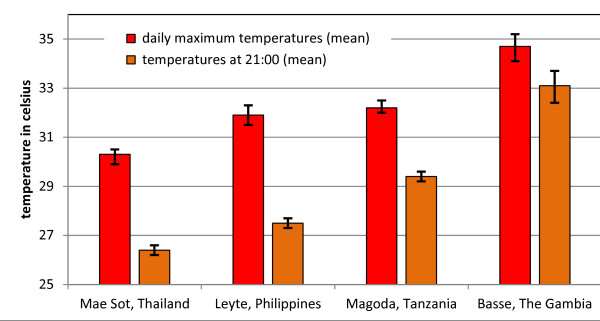
**Indoor temperatures recorded over a 12-month period by HOBO data loggers.** The hottest months of the year were identified for each site as the study period for the analysis. These were: The Gambia (February through June), Tanzania (January through April), the Philippines (April through December), and Thailand (April through October). The maximum temperature for each study house and each day during the study period was recorded and the mean daily maximum temperature for all houses in each site over the study period was calculated. To estimate the “bed time temperature” the daily temperature measurement closest to 21:00 hrs was selected for each study house and the mean temperature for all study houses over the study period was calculated.

12 months temperature recording were available from 56 of 60 houses in The Gambia (n = 12), Tanzania (n = 15), Philippines (n = 15), and Thailand (n = 14). Recordings from 4 houses were not available because the house had burned down or the dataloggers could not be retrieved for other reasons at the end of the study period. There was no statistically significant difference between recordings 50 cm and 150 cm above ground. The highest indoor temperature, 49.0 C, was recorded in the study site close to Basse Santa Su, The Gambia. The highest mean temperatures were recorded in the study sites in The Gambia (34.7°C), followed by Tanzania (32.2°C), Philippines (31.9°C), and Thailand (30.3°C). By 21:00 the temperatures had come down to a mean of 33. 1°C in the study site in The Gambia, 29.4°C in Tanzania, 27.5°C in the Philippines and 26.4°C in Thailand. (Figure 6 illustrates the mean indoor temperatures with 95% confidence intervals.)

The mean airflow outside the bed net ranged from 6 cm sec^-1^ in Tanzania to 15 cm sec^-1^ in Thailand. The bed net attenuated the airflow between 27% in the Philippines and 71% in The Gambia. Overall, the bed nets reduced airflow compared to un-attenuated airflow from 9 to 4 cm sec^-1^ or 52% (p < 0.001). No statistically significant differences in temperature or humidity were detected between the inside and the outside of the bed nets in all sites.

**Figure 7:**
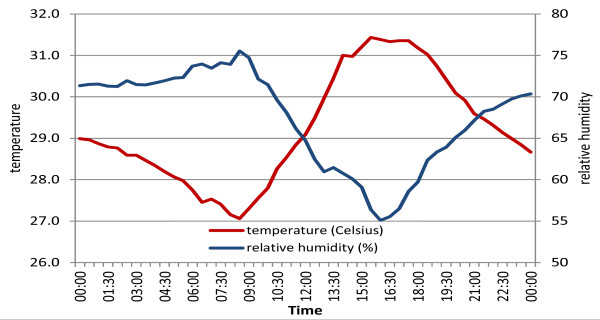
**Indoor climate during one night in a thatched mud house, Tanzania (3/03/2011; 20:30 hrs to 4/03/2011 05:30 hrs).** The temperature drops from 29.5°C at 20:30 hrs to 27.5°C the following morning. Over the same time the relative humidity increases from 69% to 74%.

**Figure 8:**
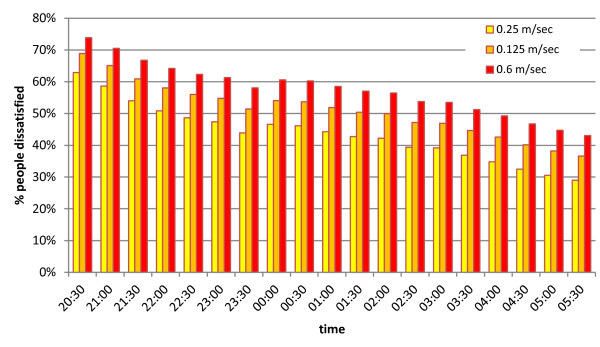
**The effect of airflow on comfort.** At 20:30 in the evening 63% of hypothetical people will feel uncomfortable despite a breeze (0.25 m/sec). The climate becomes more comfortable as the temperature drops during the night so that at 5:30 in the morning less than 30% of people will find the indoor climate uncomfortable. If we half the airflow from 0.25 to 0.125 m/sec the dissatisfaction increases and 50% of people will find it uncomfortable at 2:00 am to stay indoors. The differences in the percentage people dissatisfied between 0.25 and 0.125 m/sec and 0.125 and 0.06 m/sec are highly significant (Wilcoxon matched-pairs signed-ranks tests; p = 0.0001).

The effect of halving the airspeed is illustrated for one mud hut in Tanzania for one night in March 2011, during the hot and humid season (Figures 7 and 8). The temperature dropped from 29.5°C at 20:30 hrs to 27.5°C at 5:30 hrs the following morning while the relative humidity increased from 69% to 74% over the same period (Figure 7). At an airflow of 25 cm sec^-1^ approximately 63% of hypothetical people consider the 20:30 indoor climate uncomfortable whereas less than 30% will find the 5:30 hrs climate uncomfortable. Halving the airflow will increase the percentage of dissatisfied people to 69% at 20:30 hrs and to 37% the following morning at 5:30 hrs (Figure 8).

The wind tunnel experiments with 11 different bed nets suggested an overall reduction in airflow of 64% compared to an un-attenuated airflow with a range between 55% and 71% (Figure 5B). Nets with a mesh of 136 holes inch^-2^ reduced airflow by 55% (mean; range 51% at the highest speed up to 73% at the lowest airspeed). A denser net (mesh of 200 holes inch^2^) attenuated airflow by 59% (mean; range 56% to 74%).

A comparison of architectural characteristics relevant for thermal comfort showed marked differences between study houses in Africa and Asia (Additional file 1). The most frequently used building material for walls was mud in the African sites in contrast to bamboo and wooden planks in Asia. Eaves were open in all houses in Asia but closed in the majority of houses in Africa. The majority of houses in Asia were elevated on poles, which was not seen in Africa. The rooms used for sleeping in the African study sites had fewer – if any- windows compare to bedrooms in the Asian sites. The materials used for roofing were similar in Africa and Asia as were the use of outdoor latrines, water supply from a communal tap, and the absence of electricity in the majority of homes.

## Discussion

A commercially purchased bed net with an approximate mesh size of 220 holes inch^-2^ attenuated airflow by 52% in real life household settings. Experimental studies in a wind tunnel suggested an even more pronounced attenuation than observed in the field setting. This difference between real-life and experimental setting can be explained by the omni-directional airflow within the bed net in contrast to the unidirectional airflow in the wind tunnel. As expected the airflow was more attenuated by denser than less dense bed nets. There was no significant difference in temperature or humidity between the inside and the outside of the net. The perceived thermal discomfort reported by bed net users is therefore mainly caused by the attenuation of airflow.

Indoor temperatures in the study houses specifically in the African study sites were very high during the day time and remain high at night making comfortable sleep unlikely at least during the hottest months of the year. Installing a bed net removes the relief provided by a cooling breeze. The discomfort caused by a bed net may only be acceptable in the presence of a very high density of mosquitoes. As long as the bite rate is bearable sleeping without a bed net may be preferable for many people at risk for malaria and other vector borne diseases.

ITNs are very powerful tools to reduce malaria transmission. The results of this study support the hypothesis that thermal discomfort has limited the uptake of bed nets. Bed nets affect airflow, with little to no influence on temperature or humidity. It is likely that the effect of bed net usage on airflow would be most intolerable during the hottest and most humid period of the year, which frequently coincides with the peak of vector biting densities and malaria transmission.

Asian houses were found to be cooler and hence more comfortable than African mud huts. Elevating huts on poles assures improved airflow around all sides of the house, and elevation of homes also reduces house entry by mosquitoes [9]. The small number or absence of windows was striking in the African study sites. In contrast, the use of light, porous building materials like bamboo and wooden planks as well as open eaves, not to mention windows and doors, are likely to assure much improved airflow and comfort in the Asian houses compared to those in Africa. Based on these observations, a more sustained uptake and year-round utilization of bed nets is more likely in hot humid countries the Asia than in Africa. The current high uptake of bed nets in hot tropical Africa may not be sustainable in future if the immediate benefit of the bed net by preventing insect bites is outweighed by the discomfort caused by bed nets.

The study was not designed to provide a complete and accurate survey of the housing in the study sites nor were houses randomly selected. Instead representative houses were selected by the investigators with training in architecture. Yet the critical observations of this study, specifically the absence of elevated houses and paucity of windows in the buildings in the African sites can be probably generalized. Furthermore, the measurement of airflow could only be undertaken for a limited number of nights in 20 study houses as the thermo-anemometers are extremely fragile. It is, therefore, not possible to compare the indoor airflow between houses, which not only depends on the climate characteristics of individual buildings but also on the outdoor wind speed.

The findings suggest potential modifications in bed net design to increase their uptake. Firstly, the data show that less dense bed nets are likely to minimize the attenuation of airflow and are hence likely to be more comfortable. The largest hole size which just prevents the entry of mosquitoes is likely to be optimal to increase bed net uptake. At present, the most widely used mesh size is 156 holes inch^-2^, even though smaller vector species (like *Anopheles funestus*) are known to penetrate such nets. The difference in air flow attenuation between nets with a mesh of 136 holes per square inch (55%) and denser nets of 200 holes per square inch (59%) appears to be small overall. Hence, this mesh size may be the minimum size possible for use in malaria control even though their thermal comfort may be sub-optimal during parts of the year.

Secondly, the observations made in this study open a vista of architectural modifications for African homes, which improve airflow in the space where the bed net is employed and hence will increase the airflow within the net. Such modifications range from elevating buildings off the ground, use of more porous building materials, e.g. bamboo, or the addition of windows or doors. Finally, solar-power operated fans could be placed within bed nets to increase airflow. The distribution of high quality bed nets without assurance of thermal comfort may only provide short-term benefits.

## Competing interests

All authors have completed the ICMJE uniform disclosure form at www.icmje.org/coi_disclosure.pdf and declare no competing interests.

## Authors’ contribution

JK proposed the original study hypothesis. LvS and JK developed the project. BGJK advised on the study design. KI and RB collected the study data. MJ and MP supervised the work in The Gambia. All authors contributed to writing the manuscript. All authors read and approved the final manuscript.

## Supplementary Material

Additional file 1 Characteristics of 20 houses used for detailed airflow measurements. Click here for file

## References

[B1] Lengeler C Insecticide-treated bednets and curtains for preventing malaria The Cochrane Database of Systematic Reviews (Complete Reviews) 2004 Issue. Art. No.: CD000363 10.1002/14651858.CD000363 10796535

[B2] WHO World malaria report 2009 2009 World Health Oraganization, Geneva

[B3] Thwing J Hochberg N Vanden J Issifi S Eliades MJ Minkoulou E Wolkon A Gado H Ibrahim O Newman RD Lama M Insecticide-treated net ownership and usage in Niger after a nationwide integrated campaign Trop Med Int Health 2008 13 827 834 10.1111/j.1365-3156.2008.02070.x 18384476

[B4] Trape JF Tall A Diagne N Ndiath O Ly AB Faye J Dieye-Ba F Roucher C Bouganali C Badiane A Sarr FD Mazenot C Touré-Baldé A Raoult D Druilhe P Mercereau-Puijalon O Rogier C Sokhna C Malaria morbidity and pyrethroid resistance after the introduction of insecticide-treated bednets and artemisinin-based combination therapies: a longitudinal study Lancet Infect Dis 2011 11 925 932 10.1016/S1473-3099(11)70194-3 21856232

[B5] Pulford J Hetzel MW Bryant M Siba PM Mueller I Reported reasons for not using a mosquito net when one is available: a review of the published literature Malar J 2011 10 83 10.1186/1475-2875-10-83 21477376 PMC3080352

[B6] Koenigsberger OH Ingersoll TG Mayhew A Szokolay SV Manual of Tropical Housing and Building, Part one: Climatic Design 1974 Longman, London

[B7] Fanger P Thermal Comfort 1970 Danish Technical Press, Copenhagen

[B8] Owen SO Kennedy HE 2009 ASHRAE Handbook: Fundamentals, Prediction of thermal comfort; ASHRAE Standard 55 (chapters 6 and 8) 2005 American Society of Heating, Refrigerating and Air-Conditioning Engineers, Atlanta

[B9] Charlwood JD Pinto J Ferrara PR Sousa CA Ferreira C Gil V Do Rosário VE Raised houses reduce mosquito bites Malar J 2003 10 45 14667242 10.1186/1475-2875-2-45 PMC317347

