# Supplementary material for: Airflow attenuation and bed net utilization: observations from Africa and Asia
Source: Malar J. 2012 Jun 15;11:200. doi: 10.1186/1475-2875-11-200 (PMC3441282; doi:10.1186/1475-2875-11-200)
Supplement: Additional file 1 — Characteristics of 20 houses used for detailed airflow measurements. [file 1475-2875-11-200-S1.doc]

Additional file 1: Characteristics of 20 houses used for detailed airflow measurements

|  | **#** | Occupants | Walls | Roof | Eaves | Floor | Foundation | Indoor Animals | Number Windows | Doors | Glass Windows | Lavatory | **Water supply** | Electricity | Cooking | Age | Days To Build |
| --- | --- | --- | --- | --- | --- | --- | --- | --- | --- | --- | --- | --- | --- | --- | --- | --- | --- |
| **Thailand** | 1 | 6-10 | Wood / Concrete | Corrugate Iron | Open | Wood / Concrete | Poles | None | 8 | 3 | No | Indoor  WC | Indoor Tap | Grid | Indoor Fire | 3 Years | 45 |
|  | 2 | 3-5 | Wood / Concrete | Corrugate Iron | Open | Wood / Concrete | Concrete | None | 15 | 3 | Yes | Indoor  WC | Indoor Tap | Grid | Indoor Fire | >5 Years | ? |
|  | 3 | 6-10 | Teak Wood | Thatch | Open | Teak Wood | Concrete | None | 4 | 3 | No | Outdoor Latrine | Indoor Tap | Grid | Indoor Fire | 4 Years | 21 |
| **Philippines** | 4 | 3-5 | Nippa | Thatch | Open | Bamboo | Poles | Dog | 4 | 2 | No | Outdoor Latrine | Communal Well | No | Indoor Fire | 4 | 30 |
|  | 5 | 3-5 | Bamboo | Thatch | Open | Bamboo | Poles | Chicken | 2 | 1 | No | Outdoor Latrine | Communal Well | No | Indoor Fire | 3 | 30 |
|  | 6 | 3-5 | Bamboo | Corrugate Iron | Open | Bamboo | Poles | Dog | 5 | 2 | No | Outdoor Latrine | Communal Well | No | Indoor Fire | >5 Years | 21 |
|  | 7 | 3-5 | Bamboo | Corrugate Iron | Open | Bamboo | Poles | None | 5 | 2 | No | Outdoor Latrine | Communal Well | No | Indoor Fire | >5 Years | 60 |
|  | 8 | 3-5 | Bamboo | Corrugate Iron | Open | Bamboo | Poles | Dog | 6 | 3 | No | Outdoor Latrine | Communal Well | No | Indoor Fire | >5 Years | 90 |
|  | 9 | 6-10 | Concrete | Corrugate Iron | Open | Concrete | Concrete | None | 6 | 2 | Yes | Indoor  WC | Communal Well | Grid | Indoor Fire | 2 Years | ? |
| **The Gambia** | 10 | 2 | Mud | Corrugate Iron | Closed | Concrete | Mud Bricks | None | 0 | 2 | Na | Outdoor Latrine | Communal Well | No | Outdoor Fire | ? | ? |
|  | 11 | 2 | Mud | Corrugate Iron | Closed | Concrete | Mud Bricks | None | 0 | 2 | Na | Outdoor Latrine | Communal Well | No | Outdoor Fire | ? | ? |
|  | 12 | 2 | Mud | Thatched | Closed | Concrete | No Foundation | None | 3 | 2 | No | Outdoor Latrine | Communal Well | No | Outdoor Fire | 4 Years | 14 |
|  | 13 | 1 | Concrete | Corrugate Iron | Closed | Concrete | Concrete | None | 1 | 1 | No | Outdoor Latrine | Communal Well | No | Outdoor Fire | >5 Years | 90 |
|  | 14 | 1 | Mud | Thatched | Closed | Concrete | No Foundation | None | 0 | 2 | No | Outdoor Latrine | Communal Well | No | Outdoor Fire | >5 Years | 8 |
|  | 15 | 2 | Mud | Corrugate Iron | Closed | Concrete | Mud Bricks | No | 0 | 2 | Na | Outdoor Latrine | Communal Well | No | Outdoor Fire | >5 Years | ? |
|  | 16 | 1 | Mud | Thatched | Closed | Concrete | No | No | 0 | 2 | Na | Outdoor Latrine | Communal Well | No | Outdoor Fire | 3 | 7 |
| **Tanzania** | 17 | 3-5 | Mud | Thatched | Open | Mud | No Foundation | None | 2 | 2 | No | Outdoor Latrine | Communal Well | No | Outdoor Fire | >5 Years | 30 |
|  | 18 | 3-5 | Mud | Thatched | Closed | Mud | No Foundation | None | 4 | 2 | No | Outdoor Latrine | Communal Well | No | Indoor Fire | >5 Years | 120 |
|  | 19 | 3-5 | Mud | Thatched | Open | Concrete | No Foundation | None | 2 | 1 | No | Outdoor Latrine | Communal Well | No | Indoor Fire | >5 Years | 90 |
|  | 20 | 3-5 | Brick | Corrugate Iron | Closed | Concrete | Concrete | None | 6 | 2 | No | No Latrine | Communal Well | No | Outdoor Fire | >5 Years | ? |

WC=Water Closet = Flush Toilet, Grid= Electricity Supplied by Public Utilities, Na=Not Applicable, None of the houses had air conditioning
